# Protective Effects of Vitamin C and NAC on the Toxicity of Rifampin on Hepg_2_ Cells

**Published:** 2013

**Authors:** Nasser Vahdati-Mashhadian, Mahmoud Reza Jafari, Nasim Sharghi, Toktam Sanati

**Affiliations:** a *Department of Pharmacodynamy and Toxicology, Faculty of Pharmacy, Mashhad University of Medical Sciences, Mashhad, Iran. *; b *Biotechnology Research Center, Nanotechnology Research Center, School of Pharmacy, Mashhad University of Medical Sciences, Mashhad, Iran.*; c *Faculty of Pharmacy, Mashhad University of Medical Sciences, 91775-1365, Mashhad, Iran. *

**Keywords:** HepG2, Cell toxicity, MTT assay, Vitamin C, NAC

## Abstract

Rifampin, an antibiotic widely used for the treatment of mycobacterial infections, produces hepatic, renal and bone marrow toxicity in human and animals. In this study, the protective effects of vitamin C and *n*-acetylcysteine (NAC) on the toxicity of rifampin on HepG2 cells were investigated.

Human hepatoma cells (HepG2) were cultured in 96-well M of rifampin in the presence of microplate and exposed to 10, 20, 50 and 100 vitamin C (0.1 mg/mL) and NAC (0.2 mg/mL). Protective effect of the two drugs against rifampin toxicity was assessed by MTT assay.

Results show that both vitamin C and NAC significantly inhibited HepG2 cellular damage due to rifampin, and vitamin C was relatively more potent than NAC. Rifampin is metabolized by the liver and its toxic metabolites are responsible for the drug›s hepatic toxicity. Based on our results, it seems that reactive metabolites are the main agents responsible for rifampin hepatotoxicity. The importance of this finding is that if vitamin C or NAC do not affect the antibacterial activity of rifampin, they could be used as preventive agents in rifampin users.

## Introduction

Cell viability assays are frequently used for drug discovery using high-throughput screening (1), environmental assessment of chemicals (2) and biosensors for monitoring cellular behavior (3). Some biochemical methods, such as the MTT assay are widely used in toxicity screening assays (4, 5). 

Rifampin is an important drug in the treatment of human mycobacterial and other infections. It is widely used as an essential drug in the treatment of tuberculosis and leprosy, in combination with other drugs (6). The drug has been shown to produce hepatic toxicity in animal studies (7, 8) and clinical settings (9, 10). 

Previously in our laboratory, during the design of a cell-based test distinguishing between the toxicity of compounds per se and their toxicity resulting from their metabolites, we have shown that rifampin is toxic to human hepatoma cell line (HepG2) in concentrations in the range of its C_max_ in clinical setting (11). The drug, in agreement with clinical data, did not show any significant toxicity against human larynx carcinoma cell line (Hep2). HepG2 cells are rich in drug metabolizing enzymes (12).

Therefore, it seems that the toxicity of rifampin on HepG2 cells is due to its toxic metabolic products. In order to evaluate this hypothesis, in this study, we investigated the effects of an antioxidant, vitamin C, and a scavenger of active metabolites, NAC, on the toxicity of rifampin on HepG2 cells by MTT assay.

## Experimental

Rifampin was kindly provided by Hakim Pharmaceutical Company (Tehran, Iran). The substance was dissolved in DMSO. MTT (tissue culture grade) was purchased from Sigma (USA). DMSO and DMEM powder were purchased from Merck Company (Germany). Gentamicin was purchased as injectable ampoules from Darou Pakhsh Pharmaceutical Company (Tehran, Iran).

*Cell cultures and treatments*

HepG2 cells were obtained from Pasteur Institute Collection of Cell Cultures (Pasteur Tehran, Iran) (ECACC No. 86121112) and were cultured in DMEM supplemented with 10% FBS and gentamicin (with a final concentration of 100 mg/L) under standard conditions and subcultured in the ratio of 1:3 twice per week. Passages 1-15 were used for experiments. Cells were seeded at a density of 1000 cells/well in 96-well plates (Greiner, UK) and incubations with various concentrations of rifampin were started 24 h after seeding and continued for 48 h.

*Cytotoxicity assay*

HepG2 cells were cultured in 96-well plates at a concentration of 1 × 10^3^ cells/well, and incubated at 37ºC, in a 5% CO_2 _incubator. After 24 h, the culture supernatant was replaced with fresh DMEM and different amounts of rifampin were added to produce final concentrations of 10, 20, 50 and 100 μM in culture medium. Appropriate amounts of vitamin C and NAC were also added to produce concentrations of 0.1 and 0.2 mg/mL, respectively. The plates were incubated for 48 h. Then, 20 μL of MTT at a concentration of 5 mg/mL was added to each well. The plates were incubated for 4 h at 37ºC in a 5% CO_2_ incubator. The growth medium was removed; 200 μL of DMSO and 20 μL of glycine buffer were added and incubated at room temperature for 30 min.

The absorbance of each well was measured by an ELISA reader (Microplate reader MR 600, Dynatech, USA) at a wavelength of 570 nm. Determination of percent of growth inhibition was carried out using the following formula:

Growth inhibition (%) = [(C - T) / C] × 100

Here, C is the mean absorbance of control group and T is the mean absorbance of test group.

*Statistical evaluations*

The data were expressed as mean ± standard deviation (SD) of 8 independent experiments. Wherever appropriate, the data were subjected to statistical analysis by one-way analysis of variance (ANOVA) followed by Tukey-Kramer test for multiple comparisons. A value of p < 0.05 was considered significant. Instat 3 software was used for the statistical analysis and excel 2003 was used for producing diagrams. IC_50_ values and corresponding confidence limits (95% CL) were determined by the Litchfield and Wilcoxon method (PHARM/PCS Version 4).

## Results and Discussion

Rifampin is a valuable drug in the treatment of human tuberculosis and leprosy. A medical concern in the use of this drug is hepatotoxicity that was reported in its clinical use (9, 10). In our previous study, it was shown that rifampin produces toxicity against HepG2 cell line. This toxicity was not produced against Hep2 (Human laryngeal carcinoma) cells. It appears that phase I metabolism is responsible for the production of reactive metabolites that damage cellular macromolecules and produce lipid peroxidation (11), as these cells are rich in phase I and phase II metabolizing enzymes. They express a wide range of phase I enzymes such as cytochrome P_450_ (CYP) 1A1, 1A2, 2B, 2C, 3A and 2E1, aryl hydrocarbon hydrolase, nitroreductase, *n*-demethylase, catalase, peroxidase, NAD(P)H : cytochrome *c *reductase, cytochrome P_450_ reductase, and NAD(P)H, Quinone oxidoreductase and phase II enzymes such as epoxide hydrolase, sulfotransferase, glutathione *S*-transferase (GST), uridine glucuronosyltransferase, and *n*-acetyltransferase (12).

*Toxicity of Rifampin*

**Figure 1:**
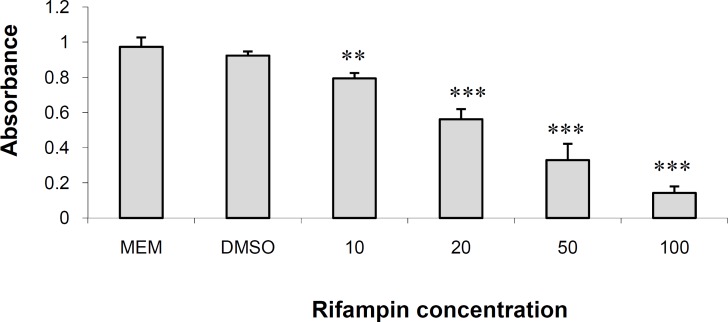
The effects of Rifampin on HepG2 cells in MTT assay.

Figure 1 shows the results of MTT assay in HepG2 cells cultured for 48 h in DMEM containing different concentrations of rifampin (see above). Rifampin produced significant dose-dependent reduction in cell number in 10 (p < 0.01), 20, 50 and 100 (p < 0.001) μM concentrations. The drug reduced the number of cells by 12.32%, 37.58%, 55.63% and 76.42% in 10, 20, 50 and 100 μM, respectively. DMSO did not reduce the number of cells to a significant amount and was not toxic to cells. The IC_50_ value for rifampin was 25.25 μM. The results are in agreement with what we and others have reported in these cells (11) and other hepatocytes (13). 

HepG2 cells (1000 cells per well) were grown in MEM for 24 h and exposed to rifampin for an additional 48 h. Rifampin concentrations are in μM. Comparisons were made with DMSO. Results presented as Mean ± SEM. MEM

(minimum essential medium) is shown to indicate the absence of a significant effect of DMSO in toxicity.

** p < 0.01 and *** p > 0.001

Rifampin is rapidly deacetylated by microsomal oxidative enzymes to the main active metabolite, 25-O-desacetylrifampin. Other metabolites of the drug are rifampin quinine, desacetylrifampin quinine, and 3-formylrifampin (14). In an animal study, rifampin altered and intensity of hepatocyte tight junctions as early as 30 min after a single dose of rifampicin (15). In another study on gel-entrapped rat hepatocytes, toxicity was related to oxidative stress and lipid accumulation, but not via cytochrome P_450_ involvement (16). They could not observe a significant toxicity in rat hepatocyte monolayer using a concentration of 12 μM of rifampin. This may suggest less sensitivity of rat hepatocyte monolayer to rifampin toxicity, compared with HepG2 cells. In addition, they stopped their experiments in 12 μM concentration, without trying higher concentrations. Besides, in our previous study, damage to cell organelles including nuclear deformation, chromosomal condensation and rough endoplasmic reticulum swelling were observed in HepG2 cells (11). Oxidative stress proposed the mechanism of toxicity in other studies as well (17).

*Experiments with vitamin C*

**Figure 2:**
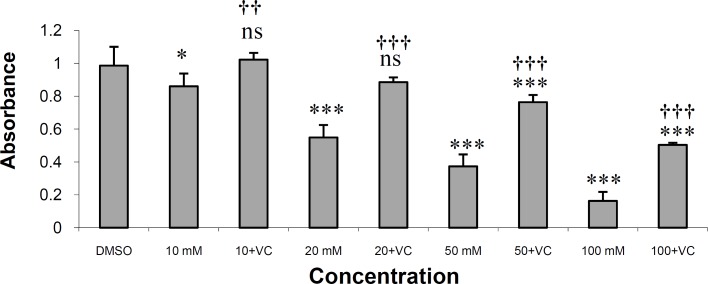
The effects of vitamin C on the toxicity of rifampin on HepG2 cells in MTT assay.

As shown in Figure 2, vitamin C at a concentration of 0.1 mg/mL effectively protected HepG2 cells against rifampin-induced toxicity. Vitamin C, at this concentration, completely protected HepG2 cells from rifampin toxicity in rifampin concentrations of 10 and 20 μM. The drug also produced significant protection in rifampin concentrations of 50 and 100 μM (p < 0.001). The IC_50 _value for rifampin is 30.52 μM and for rifampin plus vitamin C is 99.15 μM.

Vitamin C is an effective antioxidant and free radical scavenger in animal, human and *in-vitro *studies against oxidative stress and hepatotoxicity (18-20).

In this study, vitamin C could effectively protect HepG2 cells against rifampin-induced toxicity in the concentration of 0.1 mg/mL (Figure 2). 

HepG2 cells (1000 cells per well) were grown in MEM for 24 h and exposed to rifampin for an additional 48 h in the presence of vitamin C (0.1 mg/mL). Rifampin concentrations are in μM. Results presented as Mean±SEM. ns: not significant, * p < 0.05 and *** p < 0.001 for the comparison with DMSO. †† p < 0.01 and ††† p < 0.001 for the comparison of each concentration of rifampin + vitamin C (VC) with the corresponding rifampin only.

A positive aspect of the effects reported in this study is that vitamin C was effective in the concentrations in the range of its therapeutic levels (21). Some researchers reported a differential negative effect on cancer cell lines for vitamin C compared with the normal cell lines (22, 23). Considering beneficial antimicrobial-enhancing effects of vitamin C (24, 25), in addition to its antioxidant and hepatoprotective effects, makes a promising view of its potential use as a safe protective agent against rifampin-induced hepatotoxicity. Although vitamin C is not used as a prophylaxis and treatment against hepatotoxicity due to various agents, it has been shown that it is an effective prophylactic agent against oxidative stress damage to organs and tissues in animal (13,26) and human atudies (27,19).

*Experiments with NAC*

**Figure 3:**
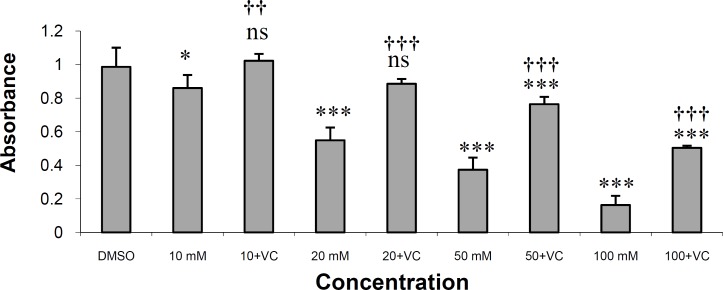
The effects of NAC on the toxicity of rifampin on HepG2 cells in MTT assay.

Figure 3 shows the result of protection of HepG2 cells from rifampin toxicity by NAC at a concentration of 0.2 mg/mL. *N-acetylcysteine *at this concentration completely protected HepG2 cells from rifampin toxicity in rifampin concentration of 10 μM. The drug also produced significant protection in rifampin concentrations of 20, 50 and 100 μM (p < 0.001). The IC_50 _value for rifampin is 25.25 μM and for rifampin plus vitamin C is 73.07 μM.

*N*-acetylcysteine is also a free-radical scavenger and hepatoprotective agent for the prevention and treatment of clinically significant liver injury in animals (28, 29) and humans (30, 31). Our results indicated that NAC effectively protected HepG2 cells against rifampin-induced toxicity at a concentration of 0.2 mg/mL (Figure 3). 

HepG2 cells (1000 cells per well) were grown in MEM for 24 h and exposed to rifampin for an additional 48 h in the presence of N-acetylcysteine (NAC, 0.2 mg/mL). Rifampin concentrations are in μM. Results presented as Mean ± SEM. ns: not significant, * p < 0.05, ** p < 0.01 and *** p < 0.001 for the comparison with DMSO. † p < 0.05 and ††† p < 0.001 for the comparison of each concentration of rifampin + NAC with the corresponding rifampin only.

NAC showed a significant protective effect against hepatotoxicity due to the administration of rifampin and isoniazid (32). In addition, Shen *et al*. showed the reduction of rifampin toxicity by ROS scavenger and NAC in gel-entrapped rat hepatocytes (16). In addition, Liang *et al*., (2010) and Daraei *et al*., (2012) found a similar effect for NAC against acetaminophen-induced cytotoxicity on human normal liver L-02 cells and depleted uranium-induced oxidative toxicity in human dermal fibroblast primary cells (33, 34). NAC is a standard drug used for the treatment of acetaminophen toxicity (30). The drug also showed protective effects against mixed antituberculosis drug-induced hepatotoxicity in a human study (35). NAC has different effects on bacterial growth. It shows antibacterial effects against P. aeruginosa and showed synergistic effects with carbenicillin or ticarcillin but antagonizes the effects of gentamicin and tobramycin on this organism (36).

In summary, both agents could effectively protect HepG2 cells against rifampin-induced toxicity with a relatively more potent effect of vitamin C compared with NAC. Both drugs showed antimicrobial-enhancing effects, although not always for NAC (25, 33). For more informative data, it is better to assess oxidative actions of rifampin using the relative biomarkers. We have no idea about the effects of these two agents on antibacterial activity of rifampin against susceptible organisms. In the case of no effect or enhancing effect, they could be promising agents (especially vitamin C because of a better safety profile) for the prevention of hepatotoxicity due to rifampin.
